# Concomitant myocardial infarction and systemic embolism associated with thrombosis of the right coronary ostium: a case report

**DOI:** 10.1093/ehjcr/ytae605

**Published:** 2024-11-15

**Authors:** Flávio Danni Fuchs, André Barcellos Amon, Aníbal Pires Borges, Felipe Costa Fuchs, Sandro Cadaval Gonçalves

**Affiliations:** Division of Cardiology, Graduate Program in Cardiology, School of Medicine, Hospital de Clínicas de Porto Alegre, Universidade Federal do Rio Grande do Sul, Ramiro Barcelos 2350, Porto Alegre, RS 90035-903, Brazil; Division of Cardiology, Graduate Program in Cardiology, School of Medicine, Hospital de Clínicas de Porto Alegre, Universidade Federal do Rio Grande do Sul, Ramiro Barcelos 2350, Porto Alegre, RS 90035-903, Brazil; Division of Cardiology, Graduate Program in Cardiology, School of Medicine, Hospital de Clínicas de Porto Alegre, Universidade Federal do Rio Grande do Sul, Ramiro Barcelos 2350, Porto Alegre, RS 90035-903, Brazil; Division of Cardiology, Graduate Program in Cardiology, School of Medicine, Hospital de Clínicas de Porto Alegre, Universidade Federal do Rio Grande do Sul, Ramiro Barcelos 2350, Porto Alegre, RS 90035-903, Brazil; Division of Cardiology, Graduate Program in Cardiology, School of Medicine, Hospital de Clínicas de Porto Alegre, Universidade Federal do Rio Grande do Sul, Ramiro Barcelos 2350, Porto Alegre, RS 90035-903, Brazil

**Keywords:** Myocardial infarction, Stroke, Concomitant, Systemic embolism

## Abstract

**Background:**

Sequential occurrences of acute ischaemic stroke in patients with acute myocardial infarction (MI) and vice versa have been reported, but not the simultaneous occurrence of both conditions. We report a case of simultaneous occurrence of MI and systemic embolism caused by a mechanism not reported to date.

**Case summary:**

A 52-year-old female patient presented with concurrent chest pain, right arm weakness, and dysphasia. An electrocardiogram demonstrated ST-elevation MI. A computed tomography angiography ruled out aortic dissection and showed an ischaemic stroke and infarction in the right kidney and spleen. A right coronary thrombotic occlusion at the ostium was successfully recanalized. Transoesophageal echocardiography showed preserved left ventricular function with no intracardiac thrombi.

**Discussion:**

The reported case presentation does not align with the mechanisms typically associated with simultaneous MI and stroke. The most plausible hypothesis is that the thrombus in the right coronary ostium extended into the aorta, resulting in a concurrent systemic embolism.

**Learning points**

- The simultaneous occurrence of an acute myocardial infarction and a stroke has been reported, but not at the exact moment.
- We documented the occurrence of a right coronary thrombotic occlusion at the ostium and evidence of systemic embolism to the brain, spleen, and kidney in a patient with concomitant presentation of acute myocardial infarction and stroke.
- Dislodging of a large thrombus in the ostium of coronary arteries to the aorta should be considered a novel cause of the simultaneous occurrence of acute myocardial infarction and systemic embolism.

## Introduction

Concurrent or sequential occurrences of acute ischaemic stroke (AIS) in patients with acute myocardial infarction (AMI) have been documented in 1.6% of patients in the USA.^1^ Interestingly, a similar incidence of 1.6% was reported for AMI in patients with AIS.^2^ These events are often referred to as concurrent or simultaneous AMI and AIS, although they typically occur sequentially, with one following the other. These occurrences can be attributed to thrombosis of coronary and cerebral vessels in the presence of pre-existing atherosclerotic disease. An event occurring in one territory can lead to clinical instability and a prothrombotic state, triggering a secondary event in another site.

Moreover, during the acute phase of myocardial infarction, thrombus formation in the left ventricular cavity can result in subsequent embolic stroke.^3^ While rare, there are instances where stroke and myocardial infarction occur simultaneously. In such cases, proposed pathophysiological mechanisms involve acute aortic dissection and simultaneous embolism affecting both coronary and cerebral arteries. Patients with atrial fibrillation may be at a heightened risk of experiencing the latter condition.^3^ Another rare condition associated with systemic embolism, including coronary arteries, is non-bacterial thrombotic endocarditis.^4^ In this report, we present a unique case of concomitant myocardial infarction and systemic embolism in a patient without any pre-existing conditions that could account for a simultaneous manifestation of these events.

## Summary figure

**Figure:**
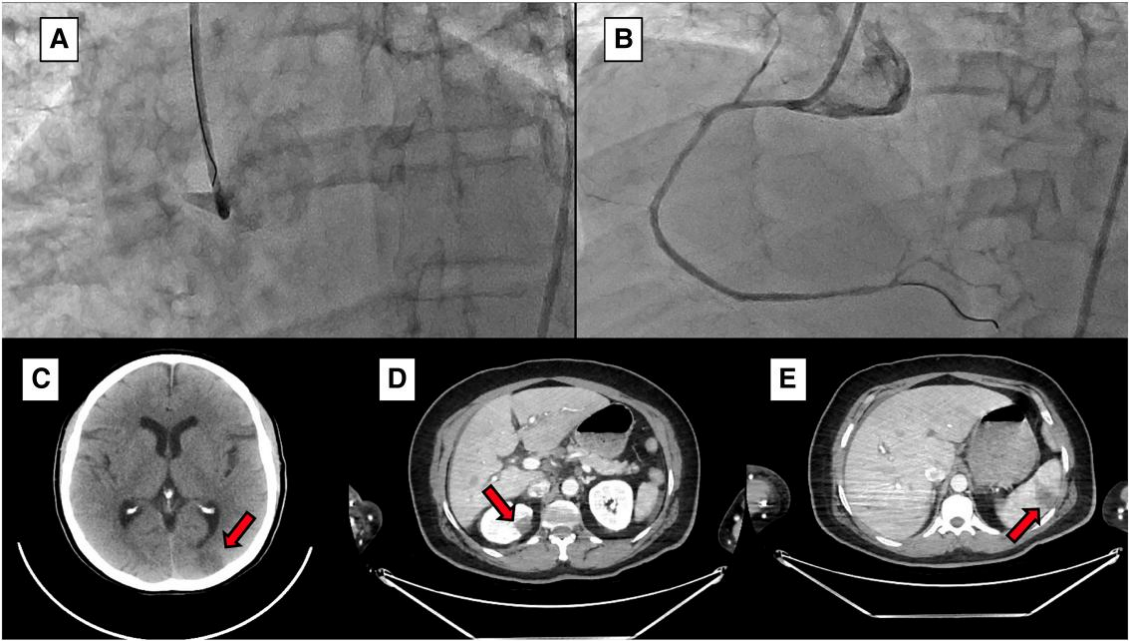
Angiographic evidence of an occluded right coronary artery at the ostium (*A*) and reperfusion after guidewire crossing of thrombus (*B*); computed tomography angiography evidence of embolism in the brain (*C*), kidney (*D*), and spleen (*E*).

## Case summary

A 52-year-old woman arrived at the emergency department with complaints of chest pain, right arm weakness, and moderate dysphasia. Her medical records revealed a history of smoking, hypertension, Type 2 diabetes, and HIV. She was on losartan for hypertension and undergoing antiretroviral treatment for HIV. Her CD4+ count was 418 cells/µL (normal 500–1500 cells/µL), and the viral load was 636 copies/mL. Diabetes was managed through dietary measures. The glycated haemoglobin was 7.2% (normal below 5.7%). There were no other relevant previous diseases on her medical record. The patient was not under other chronic treatments.

The patient reported experiencing symptoms that started simultaneously 3 h before arriving at our emergency department. The chest pain was intense, anterior, and irradiating to the left arm. The clinical presentation was corroborated by relatives who were present since the onset of the symptoms. Specifically, her son informed that at the time the patient first complained of chest pain, she was not speaking clearly, and he noticed she had lost strength in her right arm. Upon admission, the patient presented with right hemiparesis, stable vital signs, and normal cardiopulmonary examination. Electrocardiogram displayed sinus rhythm with ST-elevation in leads DII, DIII, AVF, and right precordial leads (R3R and R4R). Physical examination revealed moderate dysphasia, right hemiparesis, right-beating nystagmus, and conjugated eye deviation to the right. The remaining aspects of the physical examination yielded unremarkable findings.

**Figure 1:**
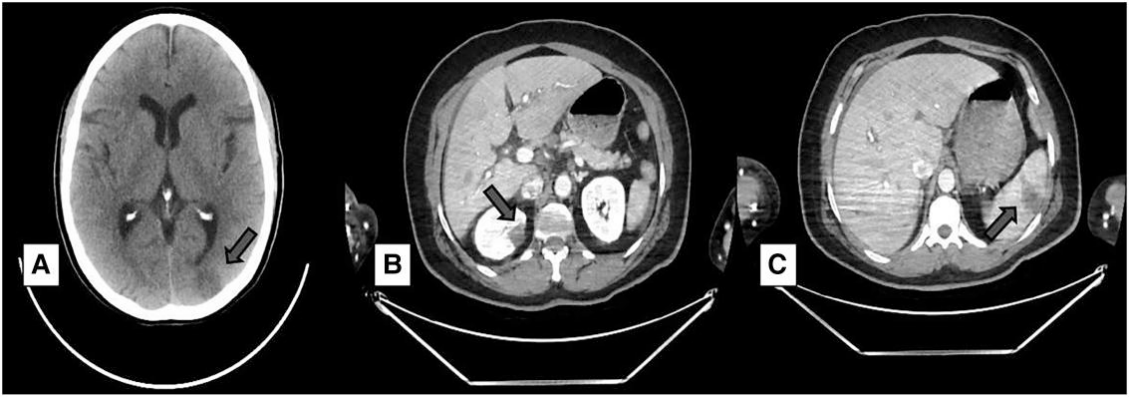
Computed tomography angiography showing embolism in the brain (*A*), kidney (*B*), and spleen (*C*).

The complexity of the presentation suggested that aortic dissection could be the origin of the cerebral and cardiac manifestations. Computed tomography angiography ruled out this hypothesis as a potential diagnosis. The exam revealed signs of an ischaemic stroke on the left parieto-occipital region, in addition to right kidney and spleen acute infarcts (*Figure 1*). The vertebrobasilar circulation and the carotid system were pervious, with no significant atherothrombotic disease. The decision to rule out the other conditions before coronary reperfusion was made since aortic dissection could explain the manifestations in the cerebral and cardiac territories.

Following consultation with a neurologist, the patient received loading doses of aspirin and clopidogrel as antiplatelet therapy. Invasive coronary angiography revealed thrombotic occlusion at the right coronary ostium (Supplementary material online, *Video S1*). The initial frame captures the occlusion observed during the diagnostic injection, followed by coronary recanalization in the second frame, restoring a normal coronary (TIMI 3) flow. Primary percutaneous coronary intervention was performed with a drug-eluting stent implantation after balloon pre-dilatation, without thrombus aspiration. Supplementary material online, *Video S1* also reveals additional atherosclerotic lesions within the right coronary artery. Additionally, non-significant lesions were observed in the left coronary artery. Additional antithrombotic therapy was initiated on the morning following the procedure with a direct oral anticoagulant (rivaroxaban).

The transthoracic echocardiogram showed a left ventricular ejection fraction of 59% with apical and inferoseptal akinesis. Transoesophageal echocardiography performed 4 days later revealed preserved global left ventricular function and apical hypokinesis a small patent foramen ovale with a mild right to left shunt, with a normal left atrium, without any intracardiac thrombi. The peak of high-sensitivity troponin was 158 000 ng/L (cut point for myocardial infarction 50 ng/L). A brain magnetic resonance revealed cortical and subcortical hyperintense signal on T2-weighted and fluid-attenuated inversion recovery sequences on the left parieto-occipital region as well as small areas on the transition of the bulb to the pons.

The patient showed favourable clinical progress from both the cardiologic and neurologic standpoints. She was seen in an outpatient consultation 1 month after discharge, with no cardiac or neurologic symptoms. The neurologic exam was normal.

A summary timeline of presentations, exams, and treatments is as follows. The simultaneous presentation of chest pain and neurologic symptoms was the index event. The patient presented at the emergency department 2 h later and underwent computed tomography angiography, which excluded aortic dissection and showed embolisms in the cerebral territory, spleen, and kidney. Following consultation with a neurologist, the patient received loading doses of aspirin and clopidogrel and underwent coronary angiography, which revealed thrombotic occlusion at the right coronary ostium (1:30 h). Subsequent exams were performed over the next 3 days. The patient was asymptomatic in an outpatient consultation 1 month after discharge.

## Discussion

We present a previously undescribed case of concomitant myocardial infarction and systemic embolism associated with thrombosis in the right coronary ostium. The diagnostic challenges posed by this concurrent presentation were significant. Initially, the possibility of acute aortic syndrome or haemorrhagic stroke was considered due to concerns regarding procedural risks and the use of anticoagulants and antiplatelet drugs in such conditions. However, as there was no evidence of aortic disease or brain haemorrhage, we proceeded with invasive coronary angiography, which revealed ostial right coronary artery obstruction.

The search for a plausible explanation for this unique clinical presentation proved perplexing. Since the patient reported the neurological manifestations at symptom onset, the possibility of a procedural complication was excluded.

The possibility that all territories suffered from systemic embolism due to thrombus from the venous territory, through the patent foramen ovale, or from the left atrium, left ventricle, or cardiac valves^3,4^ was unlikely due to the small size of the foramen, the sinus rhythm, and absence of thrombus in the left heart chambers and normal valves. However, the main evidence against systemic embolism in all territories was the coronary angiographic findings presented in Supplementary material online, *Video S1*, which unequivocally show the typical atherothrombotic occlusion of the right coronary artery ostium. The evidence of atherosclerotic disease reinforced this finding throughout the coronary territory.

Despite an exhaustive literature search, we could not find a similar case report. Taken together, these findings indicate that an ostial intracoronary thrombus that caused an AMI probably extended or dislodged into the aorta, producing simultaneous systemic embolisms.

## Conclusion

Our case represents a novel and challenging clinical scenario of simultaneous myocardial infarction and systemic embolism associated with thrombosis of the right coronary ostium. Given the lack of typical explanations, we propose that the thrombus presented at the right coronary ostium extended into the aorta, leading to concurrent systemic embolisms. These findings underscore the potential role of thrombosis at the right coronary ostium as a causative factor for systemic embolism.

## Lead author biography


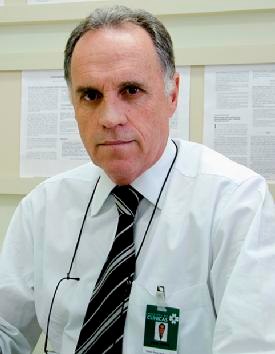


Dr Flávio Danni Fuchs is a clinical cardiologist and full professor of Cardiology at the Hospital de Clínicas de Porto Alegre, Universidade Federal do Rio Grande do Sul, Porto Alegre, Brazil. He is a senior investigator of the National Council of Research and a fellow of the American Heart Association. He has hypertension as the main area of research, employing epidemiological and clinical investigation models, including randomized controlled trials. He has over 240 manuscripts published, with more than 20,000 citations and a HI of 48.

## Supplementary Material

ytae605_Supplementary_Data

## Data Availability

The data underlying this article are available in the article and in its online supplementary material.

## Notes

**Consent:** This case report was recorded in the GPPG 89-0011 project at Hospital de Clínicas de Porto Alegre. The patient provided informed consent to have the report published anonymously.

**Funding:** None declared.
